# The value of ultrasound measurement of optic nerve sheath diameter in predicting clinical prognosis and imaging outcomes in prehospital patients with spontaneous intracerebral hemorrhage

**DOI:** 10.3389/fneur.2026.1844450

**Published:** 2026-06-22

**Authors:** Hui Jiang, Liu Yang, Jie Xiang, Zhihui Xie

**Affiliations:** 1 Department of Emergency Medical Service, The First People’s Hospital of Changde City, Changde, Hunan, China; 2 Medical Education Center of Jinan University, Guangzhou, Guangdong, China

**Keywords:** optic nerve sheath diameter, outcomes, prehospital, spontaneous intracerebral hemorrhage, survival rate

## Abstract

**Objective:**

Elevated intracranial pressure (ICP) is associated with poor prognosis in spontaneous intracerebral hemorrhage (sICH). Our study aims to assess ICP in sICH patients by measuring the optic nerve sheath diameter (ONSD) via ultrasound, and to explore whether prehospital ONSD measurement as a surrogate for ICP is associated with the clinical prognosis and imaging outcomes of sICH patients.

**Methods:**

This prospective study enrolled adult patients diagnosed with sICH upon emergency admission via prehospital care between January 2025 and December 2025. Ultrasound measurements of the ONSD were recorded at initial prehospital contact, post-departure in the ambulance, and upon hospital arrival. An ONSD ≥5 mm was defined as elevated ICP. We compared the in-hospital mortality rate, the proportion of early neurological deterioration (END), and the extent of hematoma expansion on imaging between the elevated ICP group and the normal ICP group. Regression analysis after adjusting for confounding factors was used to explore the relationship between ONSD and in-hospital mortality, END, and the extent of hematoma expansion on imaging in patients with sICH. Kaplan–Meier curves were used to analyze the survival probabilities of the two groups (ONSD ≥5 mm vs. ONSD < 5 mm).

**Results:**

A total of 186 patients were enrolled in this study, with a mean age of 62.8 ± 11.4 years. Among them, 112 patients (60.2%) were male. The ONSD ≥ 5 mm group had significantly higher rates of in-hospital mortality, END, and hematoma expansion compared to the ONSD < 5 mm group (all *p* < 0.05). After adjustment for potential confounders, ONSD increment remained independently associated with in-hospital mortality (OR: 1.35; 95% CI: 1.04–1.75; *p* = 0.024), early neurological deterioration (OR: 1.40; 95% CI: 1.08–1.89; *p* = 0.011), and hematoma expansion (OR: 1.55; 95% CI: 1.18–2.12; *p* = 0.002). Survival analysis showed that patients in the ONSD ≥ 5 mm group had a significantly lower survival rate compared to those in the ONSD < 5 mm group (*p* = 0.016).

**Conclusion:**

Among patients with acute sICH, elevated prehospital ONSD was associated with increased in-hospital mortality, END, hematoma expansion, and reduced patient survival.

## Introduction

The acute elevation of intracranial pressure (ICP) in patients with spontaneous intracerebral hemorrhage (sICH) is associated with hematoma expansion, mortality, and poor neurological outcomes (1, 2). The guidelines recommend effectively reducing intracranial pressure during the acute phase to improve survival rates, enhance neurological function, and limit hematoma expansion (3).

Elevated intracranial pressure is associated with hematoma expansion and aggravated neurological deficits. Current data and experience regarding ICP management in acute sICH patients are primarily derived from in-hospital settings (4–7). However, since most hematoma expansion occurs within 3 h of symptom onset, prehospital management of ICP during the acute phase in sICH patients directly impacts neurological outcomes (8, 9). Currently, objective prehospital assessment of ICP for acute sICH patients remains at an empirical treatment stage, lacking supporting objective data.

Ultrasound measurement of the optic nerve sheath diameter (ONSD), as a non-invasive surrogate for ICP, has been proven to correlate well with intracranial pressure in neurocritical patients and is widely promoted due to its suitability for the prehospital environment (10–13). However, whether prehospital assessment of ICP levels via ultrasound measurement of ONSD in sICH patients may help evaluate clinical prognosis and be associated with imaging outcomes remains unclear.

This study aims to explore this issue, providing data to support the clinical value of prehospital ultrasound measurement of ONSD as a noninvasive surrogate for assessing ICP levels in sICH patients.

## Materials and methods

### Study design

Patients with suspected stroke who were admitted to our hospital’s pre-hospital emergency department between January 2025 and December 2025 were prospectively enrolled. They underwent emergency computed tomography (CT) scanning upon admission and were diagnosed with sICH. The researcher obtained approval from the Ethics Review Committee of the First People’s Hospital of Changde City (Ethics Approval Number: YX-2025-517-01, approval date: January 2, 2025) and obtained informed consent from the patients or their families.

### Participant selection criteria

Inclusion criteria were as follows: (1) age >18 years; (2) CT-confirmed non-traumatic intracerebral hemorrhage meeting the diagnostic criteria (14); and (3) onset within 24 h. Exclusion criteria included: (1) cerebral venous thrombosis; (2) intracerebral hemorrhage caused by tumors or structural vascular abnormalities; (3) isolated intraventricular hemorrhage; (4) patients who underwent initial CT more than 24 h after symptom onset; (5) ocular trauma; (6) history of ocular diseases such as glaucoma, optic neuropathy, or intracranial space-occupying lesions; and (7) unequal pupil size.

### Data collection

The following data were collected: age, sex, and clinical information related to intracerebral hemorrhage, including neurological and functional assessments. The National Institutes of Health Stroke Scale (NIHSS) score at admission, Glasgow Coma Scale (GCS) score at admission, modified Rankin Scale (mRS) scores before admission and at discharge, blood pressure control, osmotherapy, surgical intervention, ICU admission, anticoagulation reversal, and hemostatic therapy were recorded.

### Imaging

Hematoma size on head CT was assessed by two experienced radiologists. The extent of the hematoma was compared between the initial head CT at admission and the head CT performed within 3 days after admission. The evaluation of both the initial and follow-up CT scans included three aspects: the location of the hematoma, the volume of bleeding, and the presence of intraventricular extension. If multiple hematomas were present, the largest one was considered.

The hematoma volume on both the initial and follow-up CT scans was estimated using the following formula: maximum length × maximum width × slice thickness × number of slices containing hematoma/2. Subsequently, the hematoma volume measurements were verified using MIStar software, which also provided a more precise volumetric assessment.

### ONSD measurement

ONSD measurements were performed by two physicians, each with 3 years of ultrasound experience. With the patient in the supine position and the head kept in a neutral alignment, the eyelids were gently closed and the eyes were kept as still as possible. Both eyes were protected using a single-use transparent dressing. A 14–5 MHz linear ultrasound probe (CX50, Philips, USA) was placed lightly on the closed upper eyelid without applying pressure to the eyeball.

Scans were performed in both transverse and sagittal planes. For the transverse scan, the probe was positioned horizontally on the closed upper eyelid; for the sagittal scan, it was positioned vertically. The angle was adjusted to obtain the optimal image of the optic nerve sheath on both sides. Measurements were taken at 3 mm behind the globe.

To minimize measurement error, ONSD was measured three times in each eye, resulting in a total of six measurements recorded as one set. The average of these six measurements was calculated as the subject’s ONSD value, reported to an accuracy of 0.01 mm.

A recent systematic review and meta-analysis by Berhanu et al. (15) reported that the weighted median optimal ONSD cut-off for detecting increased intracranial pressure was 5.3 mm (IQR: 4.9–5.6 mm), and 10 studies used a predefined cut-off of 5.0 mm. Based on these findings and our own ROC analysis (Supplementary Figure S1), we selected 5 mm as a practical and clinically feasible threshold for prehospital risk stratification. For the main analyses, the mean ONSD of the three prehospital measurements was calculated and used for group allocation and all regression analyses.

### Outcomes

The primary outcomes observed were in-hospital mortality, early neurological deterioration (END), and imaging evidence of increased hematoma extent. END was defined as an increase in the NIHSS score by ≥4 points within 72 h of admission (16). Imaging evidence of increased hematoma extent was defined as an increase in hematoma volume by 6 mL and/or by 33% from the initial to follow-up CT (17). The secondary outcomes included the mRS at discharge, death or dependency at discharge (defined as mRS score of 3–6), and death at 30 days.

### Statistical analysis

Data were analyzed using SPSS software (version 20.0). Continuous variables were expressed as mean ± SD when normally distributed or median (25–75%, interquartile range) when non-normally distributed, and categorical variables were expressed as frequencies and percentages. Comparisons between ONSD ≥5 mm and ONSD <5 mm were performed using independent-samples *t*-test or Mann–Whitney *U* test. The chi-square test or Fisher’s exact test was used to compare the categorical variables. A Bland–Altman analysis was performed to determine interobserver reliability.

A multivariate regression analysis model was established to adjust for potential confounding factors, including age, sex, history of diabetes, use of antiplatelet, anticoagulant, and antihypertensive medications, GCS score on admission, pre-stroke mRS score, admission NIHSS score, initial hematoma volume, and presence of intraventricular hemorrhage. Additionally, we included important acute management variables that influence sICH outcomes, including blood pressure control, osmotherapy, surgical intervention, intensive care admission, anticoagulation reversal, and hemostatic therapy. By constructing a multivariate regression model that controlled for these confounding factors, the relationship between pre-hospital ONSD, analyzed as a continuous variable in 0.5 mm increments, and in-hospital mortality, END, and increased hematoma extent was examined. Kaplan–Meier curves were used to analyze the survival probabilities of the two groups (ONSD ≥5 mm vs. ONSD < 5 mm).

To assess the incremental prognostic value of ONSD beyond established clinical parameters, we constructed two logistic regression models for each outcome:

Model A (baseline model): included conventional clinical predictors, including age, sex, admission GCS score, admission NIHSS score and initial hematoma volume.

Model B (full model): included all variables in Model A plus ONSD as a continuous variable.

Receiver operating characteristic (ROC) curves were generated to visually compare the discriminatory ability of the two models. Model performance was compared using the area under the ROC curve (AUC), with statistical significance assessed by the DeLong test. A two-tailed *p* < 0.05 was considered statistically significant.

## Results

### Patient characteristics

**Figure 1:**
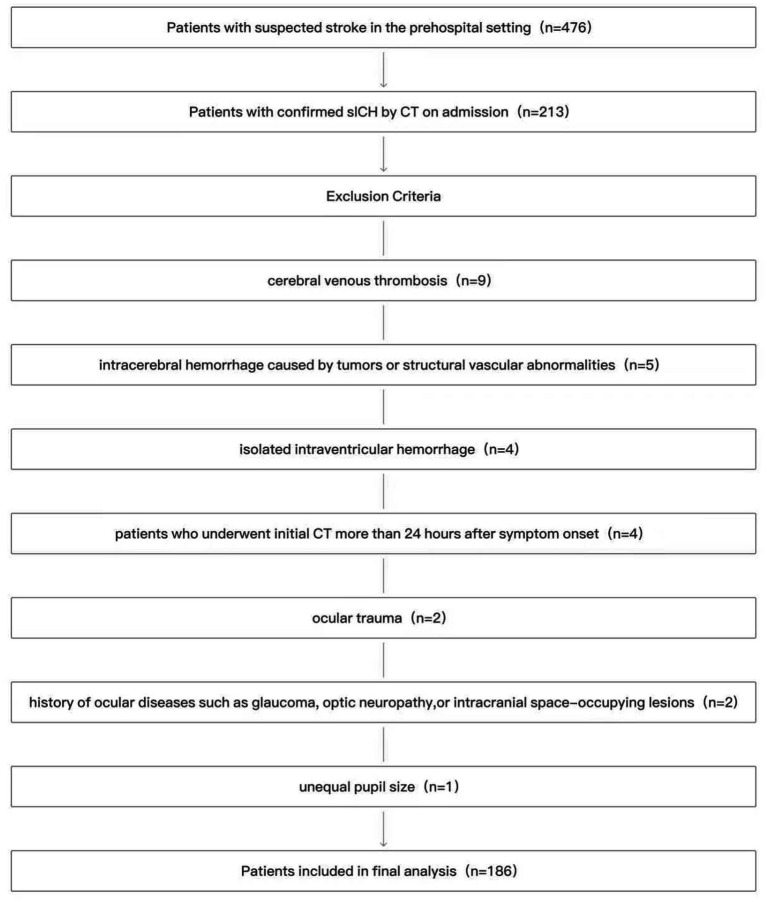
Flow chart of research.

During the study period, a total of 476 patients with suspected stroke were admitted in the pre-hospital setting, of which 186 met the inclusion criteria for this study (Figure 1). In the ONSD ≥ 5 mm group, there were 88 cases, and in the ONSD < 5 mm group, there were 98 cases. There were no statistically significant differences between the two groups in terms of age, sex, medical history (such as hypertension, diabetes, and history of antiplatelet or anticoagulant use), and hematoma location (*p* > 0.05). Compared with the ONSD < 5 mm group, patients in the ONSD ≥ 5 mm group had lower GCS scores, higher NIHSS scores, larger initial CT hematoma volume, and a higher proportion of intraventricular hemorrhage on admission, with statistically significant differences (*p* < 0.05) (Table 1).

**Table 1:** Baseline characteristics for the 186 included patients.

	Total (*n* = 186)	ONSD < 5 mm (*n* = 98)	ONSD ≧ 5 mm (*n* = 88)	*p*
Age (*y*, *x̅* ± *s*)	62.8 ± 11.4	61.9 ± 10.8	63.8 ± 12.1	0.249
Male (*n*, %)	112 (60.2)	58 (59.2)	54 (61.4)	0.762
Medical history (*n*, %)				
Hypertension	142 (76.3)	74 (75.5)	68 (77.3)	0.779
Diabetes	48 (25.8)	24 (24.5)	24 (27.3)	0.666
Antiplatelet or anticoagulant use	36 (19.4)	18 (18.4)	18 (20.5)	0.721
Clinical assessment on admission				
GCS [M (P25, P75)]	12 (8, 15)	14 (11, 15)	9 (6, 12)	<0.001
NIHSS [M (P25, P75)]	14 (8, 22)	10 (6, 15)	19 (14, 26)	<0.001
Initial CT imaging				
Hematoma location (*n*, %)				0.279
Lobar	102 (54.8)	58 (59.2)	44 (50.0)	
Deep	38 (20.4)	18 (18.4)	20 (22.7)	
Infratentoria	32 (17.2)	16 (16.3)	16 (18.2)	
Nonclassifiable	14 (7.5)	6 (6.1)	8 (9.1)	
hematoma volume (mL, *x̅* ± *s*)	32.6 ± 18.4	24.3 ± 12.6	41.8 ± 19.2	<0.001
Presence of intraventricular blood on initial CT (*n*, %)	78 (41.9)	28 (28.6)	50 (56.8)	<0.001

GCS, Glasgow Coma Scale; NIHSS, National Institutes of Health Stroke Scale.

### Reliability of measurements

A Bland–Altman analysis yielded a mean (SD) difference of 0.03 (0.22) mm in measurements between the two observers. For hematoma volume measurements, Bland–Altman analysis demonstrated a mean (SD) difference of 0.5 (1.5) mL between the two radiologists.

### Clinical outcomes

Compared with the ONSD < 5 mm group, the ONSD ≥ 5 mm group had significantly higher rates of in-hospital mortality (*p* = 0.017), END (*p* = 0.023), and hematoma expansion (*p* < 0.001). In addition, patients in the ONSD ≥ 5 mm group had significantly higher median mRS scores at discharge (*p* = 0.013), as well as higher rates of poor functional outcome at discharge (mRS score 3–6) (*p* < 0.001) and 30-day mortality (*p* = 0.012), compared to those in the ONSD < 5 mm group (Table 2).

**Table 2:** Comparison of clinical outcomes between the ONSD < 5 mm and the ONSD ≥ 5 mm.

	Total (*n* = 186)	ONSD<5 mm (*n* = 98)	ONSD≧5 mm (*n* = 88)	*p*
Primary outcomes				
In-hospital mortality (*n*, %)	42 (22.6)	12 (12.2)	30 (34.1)	0.017
Early neurological deterioration (*n*, %)	58 (31.2)	18 (18.4)	40 (45.5)	0.023
Hematoma expansion (*n*, %)	64 (34.4)	20 (20.4)	44 (50.0)	<0.001
Secondary outcomes				
mRS at discharge [M (P25, P75)]	4 (2, 6)	3 (1, 4)	5 (4, 6)	0.013
Death or dependency at discharge (*n*, %)	124	48 (49.0)	76 (86.4)	<0.001
30 d mortality (*n*, %)	59 (31.7)	25 (25.5)	34 (38.6)	0.012

mRS, modified Rankin scale.

### Multivariable regression analysis of the association between ONSD and clinical outcomes

In the unadjusted logistic regression analysis, each 0.5 mm increase in ONSD was significantly associated with an increased risk of in-hospital mortality (OR: 1.68; 95% CI: 1.32–2.14; *p* < 0.001), early neurological deterioration (OR: 1.74; 95% CI: 1.38–2.19; *p* < 0.001), and hematoma expansion (OR: 2.86; 95% CI: 1.58–5.18; *p* < 0.001).

After adjustment for potential confounders, each 0.5 mm increase in ONSD remained significantly associated with in-hospital mortality (OR: 1.35; 95% CI: 1.04–1.75; *p* = 0.024), END (OR: 1.40; 95% CI: 1.08–1.89; *p* = 0.011), and hematoma expansion (OR: 1.55; 95% CI: 1.18–2.12; *p* = 0.002) (Table 3).

**Table 3:** Multivariable logistic regression analyses of prehospital ONSD and primary outcomes.

Clinical outcomes		ONSD per 0.5 mm increment	*p*
In-hospital mortality	Unadjusted logistic regression, OR (95%CI)	1.68 (1.32–2.14)	<0.001
	Adjusted analysis, OR (95%CI)	1.35 (1.04–1.75)	0.024
Early neurological deterioration	Unadjusted logistic regression, OR (95%CI)	1.74 (1.38–2.19)	<0.001
	Adjusted analysis, OR (95%CI)	1.40 (1.08–1.89)	0.011
Hematoma expansion	Unadjusted logistic regression, OR (95%CI)	2.86 (1.58–5.18)	<0.001
	Adjusted analysis, OR (95%CI)	1.55 (1.18–2.12)	0.002

### Survival analysis

**Figure 2:**
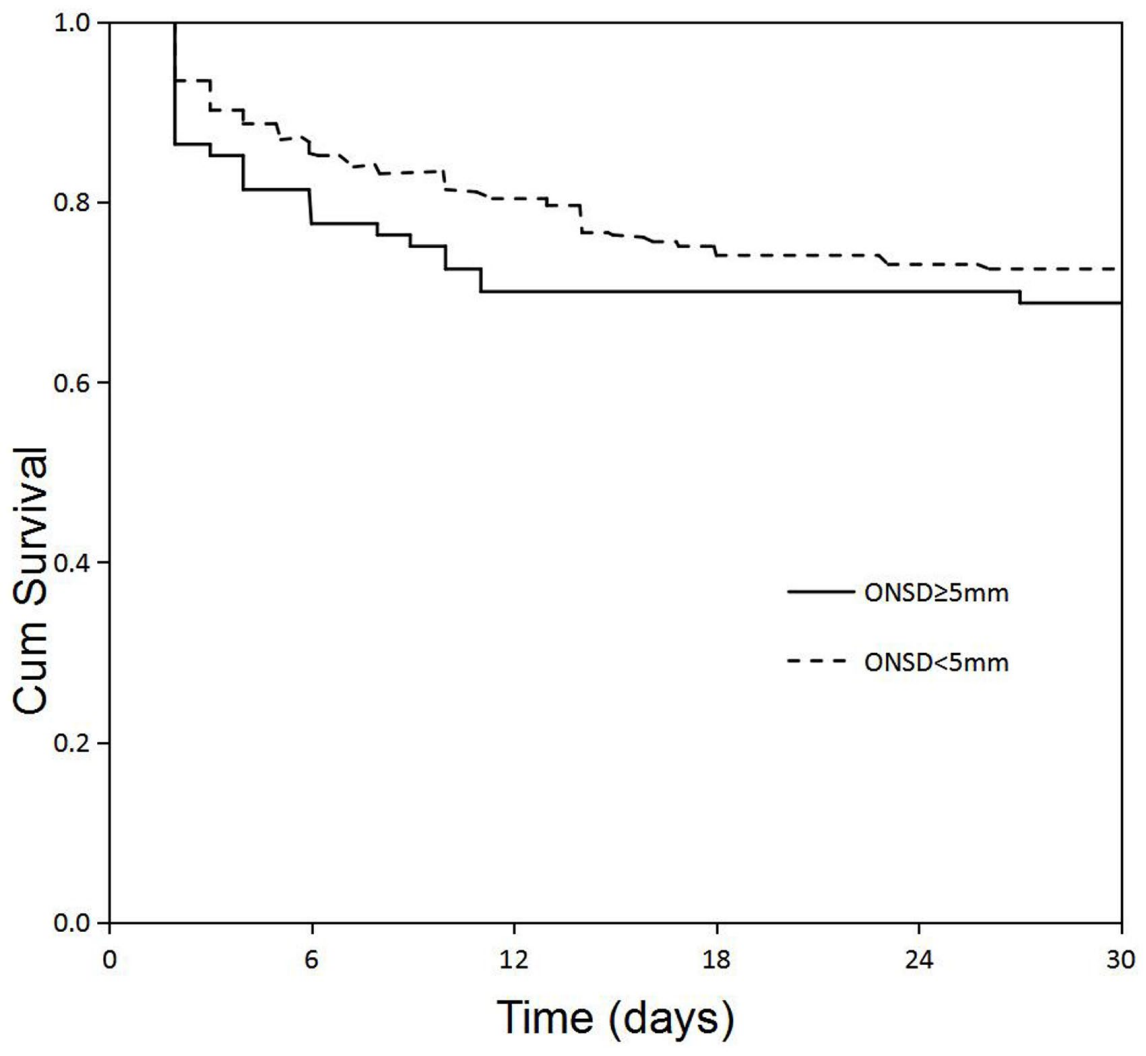
Kaplan–Meier curves of 30-day survival for patients with ONSD ≥5 mm versus ONSD <5 mm.

The 30-day survival curve demonstrated that sICH patients with prehospital ONSD ≥5 mm had a significantly shorter survival time compared to those with ONSD <5 mm, as determined by the log-rank test (*χ*^2^ = 10.332, *p* = 0.016) (Figure 2).

### Incremental prognostic value of ONSD

To determine whether adding ONSD improved predictive performance beyond conventional clinical parameters, we compared Model A (without ONSD) and Model B (with ONSD). As shown in Table 4, the addition of ONSD significantly improved the AUC for all three outcomes. For in-hospital mortality, the AUC increased from 0.74 (95% CI: 0.66–0.82) to 0.79 (95% CI: 0.72–0.86) (*p* = 0.023). For END, the AUC increased from 0.71 (95% CI: 0.63–0.79) to 0.76 (95% CI: 0.68–0.84) (*p* = 0.031). For hematoma expansion, the AUC increased from 0.73 (95% CI: 0.65–0.81) to 0.78 (95% CI: 0.71–0.85) (*p* = 0.018).

**Table 4:** Model performance comparison with and without ONSD.

Outcome	Model	AUC (95% CI)	*p*
In-hospital mortality	A (without ONSD)	0.74 (0.66–0.82)	0.023
	B (with ONSD)	0.79 (0.72–0.86)	
END	A (without ONSD)	0.71 (0.63–0.79)	0.031
	B (with ONSD)	0.76 (0.68–0.84)	
Hematoma expansion	A (without ONSD)	0.73 (0.65–0.81)	0.018
	B (with ONSD)	0.78 (0.71–0.85)	

## Discussion

sICH is a major cause of mortality and disability worldwide (18). Following the onset of sICH, blood extravasates into and compresses the brain parenchyma, leading to an increase in intracranial volume and subsequent elevation of ICP. Therefore, reducing ICP is a critical component in the management of sICH (19, 20), and the prehospital phase often represents the first window for intervention. Prehospital assessment and management of ICP are essential for improving survival rates in patients with sICH. However, studies investigating the association between prehospital assessment of ICP and clinical outcomes or radiological progression in patients with sICH remain limited. The present study was conducted to address this gap.

Given the limited equipment available for prehospital assessment of ICP in patients with sICH, invasive ICP monitoring cannot be performed in ambulances. Moreover, most patients present with varying degrees of consciousness disturbance, which further increases the difficulty of prehospital management. Considering these factors, the present study adopted ultrasound measurement of the ONSD as a method for prehospital assessment of ICP levels in patients with sICH. This technique is characterized by bedside applicability, convenience, high efficiency, and reproducibility, making it suitable for prehospital emergency settings. Previous studies by international researchers have also validated the feasibility of using ultrasound-measured ONSD for prehospital ICP assessment, suggesting its ability to accurately reflect ICP levels in patients (21, 22).

A total of 186 patients with acute spontaneous intracerebral hemorrhage were enrolled in this study. The results showed that increased prehospital ONSD was significantly associated with in-hospital mortality, END, and radiological hematoma expansion. These findings are consistent with previous reports indicating that elevated intracranial pressure during the acute phase of sICH is associated with poor prognosis (23). Regarding secondary outcomes, patients in the ONSD ≥5 mm group had worse neurological outcomes at discharge, with significantly higher rates of poor prognosis and 30-day mortality compared with the control group, further confirming the predictive value of prehospital ONSD for long-term outcomes. In addition, this study analyzed ONSD as a continuous variable in increments of 0.5 mm, allowing for a more refined quantification of the dose–response relationship between ONSD and clinical outcomes, thereby providing a more operationally applicable reference threshold for clinical practice.

The optic nerve is encased by the optic nerve sheath, and the space between the optic nerve and its sheath communicates with the subarachnoid space. The optic nerve sheath itself possesses elastic properties, and the surrounding complex structure, composed of collagen fibers, is capable of bending, contracting, and stretching, exhibiting elastic characteristics. When intracranial pressure increases, the optic nerve sheath and the surrounding elastic structures expand, leading to an increase in ONSD. Ultrasound measurement of ONSD, as one of the representative noninvasive intracranial pressure monitoring techniques, was applied in prehospital patients with sICH in this study, further validating the close correlation between ONSD and intracranial pressure. The results showed that each 0.5 mm increase in ONSD was an independent risk factor for in-hospital mortality, END, and hematoma expansion in patients with sICH. Multivariable regression analysis revealed that after adjusting for potential confounders, the associations between each 0.5 mm increase in ONSD and in-hospital mortality (OR: 1.35, *p* = 0.024), END (OR: 1.40, *p* = 0.011), and hematoma expansion (OR:1.55, *p* = 0.002) remained statistically significant. Similarly, Elveren et al. (24) demonstrated that ONSD correlates with clinical outcomes in patients undergoing decompressive craniectomy for cerebral infarction, but noted that this correlation largely reflects disease severity. Consistent with the findings, our results suggest that ONSD should be interpreted as one component of a multifactorial risk assessment approach rather than a standalone predictor.

The use of ultrasound-measured ONSD to assess intracranial pressure has not only been validated in patients with sICH but has also been widely confirmed for its accuracy and practicality in other conditions within the field of neurocritical care, including subarachnoid hemorrhage, traumatic brain injury, brain tumors, and cerebral vascular malformations (25, 26). Furthermore, some researchers have proposed that regardless of the final diagnosis, ultrasound measurement of ONSD may be independently associated with intracranial hypertension (27), indicating that the predictive value of ONSD for intracranial pressure levels is independent of disease type, demonstrating good generalizability and clinical applicability.

The results of this study demonstrate that in patients with sICH, an increase in ONSD is associated with worsening outcomes in three key indicators of neurological prognosis: in-hospital mortality, END, and hematoma expansion. These findings suggest that assessment and management of intracranial pressure during the early phase of the disease are critically important for the treatment of patients with sICH. Bedside ultrasound measurement of ONSD to assess intracranial pressure fills a gap in effective noninvasive ICP assessment during the prehospital phase. The survival analysis in this study revealed that patients in the ONSD ≥5 mm group had significantly shorter survival times compared with those in the ONSD <5 mm group, further emphasizing the importance of prehospital ICP evaluation using ONSD in patients with sICH. Importantly, our model comparison analyses demonstrated that adding ONSD to conventional clinical parameters (GCS, NIHSS and hematoma volume) significantly improved predictive performance for all three outcomes. These findings suggest that ONSD provides incremental prognostic value beyond established clinical parameters, supporting its potential role as an adjunctive tool for early risk stratification in prehospital sICH patients.

A recent scoping review by Martínez-Palacios et al. (28) comprehensively evaluated the use of ONSD for ICP monitoring in traumatic brain injury, highlighting substantial methodological heterogeneity across studies and noting that ONSD as a standalone tool has important limitations. Our findings are consistent with this review, as we observed a modest incremental value when adding ONSD to conventional clinical parameters. These results support the notion that ONSD should be used as an adjunctive tool rather than a standalone predictor, and underscore the need for standardized measurement protocols in future prehospital research.

Currently, prehospital assessment of intracranial pressure is largely based on clinical signs such as blood pressure and pupillary examination. Due to the lack of objective and accurate methods for intracranial pressure assessment, along with limited guidance from guidelines and consensus statements regarding indications for prehospital intracranial pressure intervention, the indications for prehospital intracranial pressure management remain unclear, and treatment is often empirical. Ultrasound measurement of ONSD offers high accuracy in assessing intracranial pressure in neurocritical care patients and can be widely implemented in the prehospital setting. Therefore, this noninvasive technique provides an objective solution to the challenges of empirical assessment of intracranial pressure levels and the ambiguity of intervention indications in prehospital patients with sICH. The findings of this study suggest that for patients with ONSD ≥5 mm, close monitoring of dynamic changes in ONSD values and timely implementation of effective intracranial pressure-lowering strategies may help improve patient survival.

This study has several limitations. First, it was a single-center prospective observational study with a relatively limited sample size, which may introduce selection bias; thus, the generalizability of the findings needs to be further validated by multicenter studies with larger sample sizes. Second, the measurement of ONSD by ultrasound involves a certain degree of subjectivity. Although measurements were performed by physicians who underwent standardized training, and the average of multiple measurements from both eyes was used to minimize error, inter-operator variability cannot be completely eliminated. Third, ONSD was assessed at only three time points during the prehospital phase, and continuous dynamic monitoring was not achieved. Finally, due to the observational design of this study, the identified associations between ONSD and clinical outcomes should be interpreted as correlational rather than causal.

## Conclusion

Ultrasound measurement of ONSD is a reliable and reproducible noninvasive technique for assessing intracranial pressure. It may help reflect intracranial pressure levels in prehospital patients with sICH and could provide an objective basis for early identification of patients at higher risk of poor clinical outcomes and radiological progression during the prehospital phase.

## Data Availability

The original contributions presented in the study are included in the article/Supplementary material, further inquiries can be directed to the corresponding author.
