# Supplementary figures and images for: The value of ultrasound measurement of optic nerve sheath diameter in predicting clinical prognosis and imaging outcomes in prehospital patients with spontaneous intracerebral hemorrhage
Source: Front Neurol. 2026 Jun 22;17:1844450. doi: 10.3389/fneur.2026.1844450 (PMC13333521; doi:10.3389/fneur.2026.1844450)

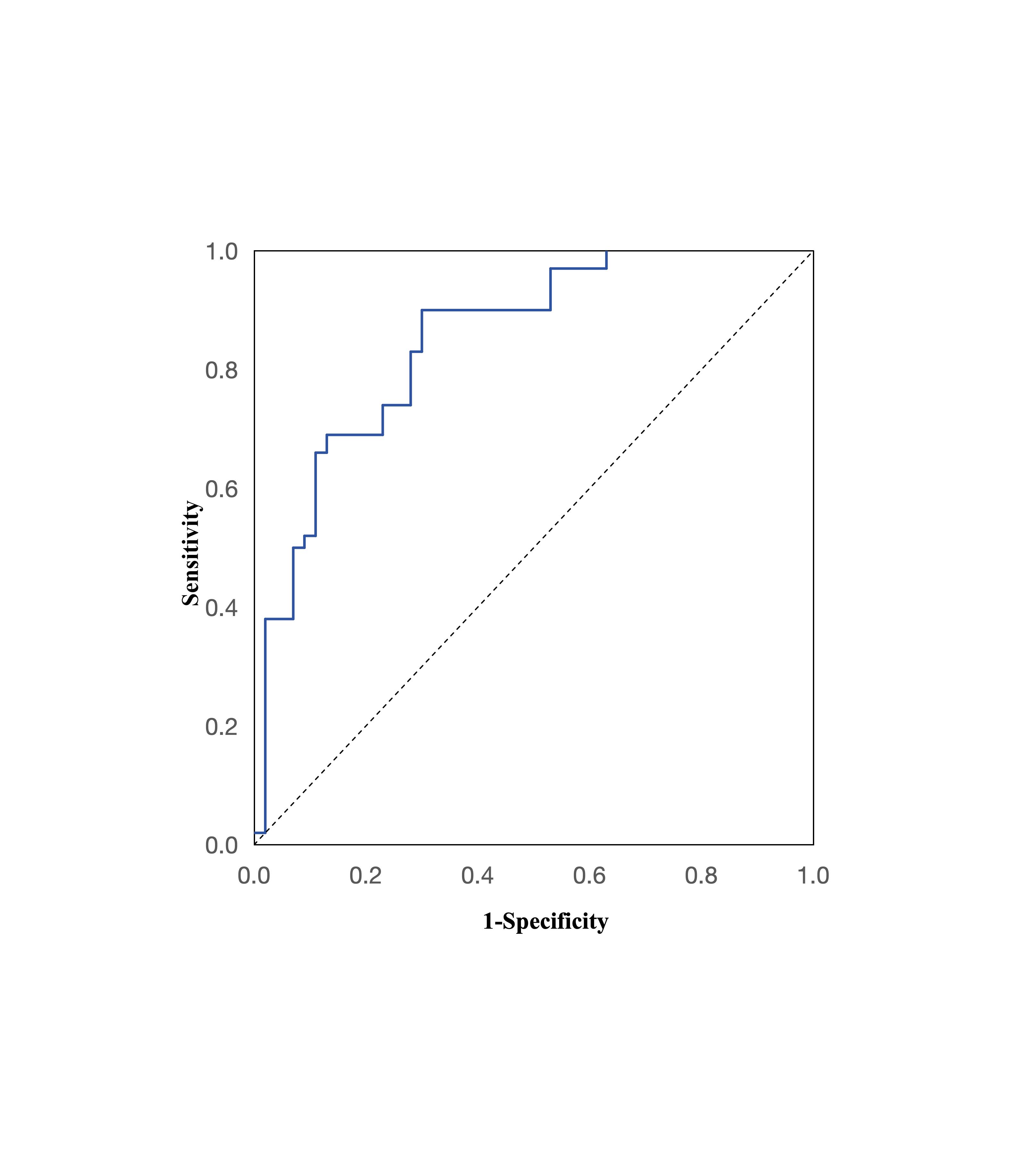

Supplement: Supplementary file 1 [file Image_1.jpeg]
